# Interpretation of spectroscopic data using molecular simulations for the secondary active transporter BetP

**DOI:** 10.1085/jgp.201812111

**Published:** 2019-03-04

**Authors:** Vanessa Leone, Izabela Waclawska, Katharina Kossmann, Caroline Koshy, Monika Sharma, Thomas F. Prisner, Christine Ziegler, Burkhard Endeward, Lucy R. Forrest

**Affiliations:** 1 Computational Structural Biology Section, National Institutes of Neurological Disorders and Stroke, National Institutes of Health, Bethesda, MD; 2 Max Planck Institute for Biophysics, Frankfurt am Main, Germany; 3 Institute of Biophysics and Biophysical Chemistry, University of Regensburg, Regensburg, Germany; 4 Institute of Physical and Theoretical Chemistry and Center of Biomolecular Magnetic Resonance, Goethe University, Frankfurt am Main, Germany

## Abstract

Mechanistic understanding of dynamic membrane proteins such as transporters, receptors, and channels requires accurate depictions of conformational ensembles, and the manner in which they interchange as a function of environmental factors including substrates, lipids, and inhibitors. Spectroscopic techniques such as electron spin resonance (ESR) pulsed electron–electron double resonance (PELDOR), also known as double electron–electron resonance (DEER), provide a complement to atomistic structures obtained from x-ray crystallography or cryo-EM, since spectroscopic data reflect an ensemble and can be measured in more native solvents, unperturbed by a crystal lattice. However, attempts to interpret DEER data are frequently stymied by discrepancies with the structural data, which may arise due to differences in conditions, the dynamics of the protein, or the flexibility of the attached paramagnetic spin labels. Recently, molecular simulation techniques such as EBMetaD have been developed that create a conformational ensemble matching an experimental distance distribution while applying the minimal possible bias. Moreover, it has been proposed that the work required during an EBMetaD simulation to match an experimentally determined distribution could be used as a metric with which to assign conformational states to a given measurement. Here, we demonstrate the application of this concept for a sodium-coupled transport protein, BetP. Because the probe, protein, and lipid bilayer are all represented in atomic detail, the different contributions to the work, such as the extent of protein backbone movements, can be separated. This work therefore illustrates how ranking simulations based on EBMetaD can help to bridge the gap between structural and biophysical data and thereby enhance our understanding of membrane protein conformational mechanisms.

## Abstract (precis)

Use of spectroscopic techniques to complement structural data and enhance mechanistic understanding of membrane proteins is often complicated by experimental discrepancies. Leone et al. address this challenge by applying an advanced simulation methodology to the interpretation of spectroscopic data for the transporter protein BetP.

## Introduction

Molecular mechanisms of signaling, solute transport, and permeation across membranes typically require a membrane protein to undergo one or more conformational changes. For example, in active transport, in which a solute is moved against its concentration gradient, the transporter must expose the substrate binding site to either side of the membrane in a process known as alternating access (Jardetzky, 1966; Mitchell, 1967). Coupling the conformational changes to binding of sodium, for example, moving along a preexisting concentration gradient, energizes the transport process, resulting in net accumulation of the substrate. Coupling ions, substrates, and other environmental factors such as the lipid composition can all affect these conformational equilibria. Understanding how these factors act on membrane proteins during alternating access requires an atomistic description of the conformational ensembles; only then can the underlying energy landscapes, and the shifts therein, be accurately described (Faraldo-Gómez and Forrest, 2011; Masureel et al., 2014; Liao et al., 2016; Ruan et al., 2017).

The past couple of decades have seen major successes in structural biology, revealing architectures and major conformational states of a wide range of transporters, channels, and receptors (Boudker and Verdon, 2010; Forrest et al., 2011; Manglik and Kobilka, 2014; Yan, 2015; Ahern et al., 2016; Drew and Boudker, 2016; Goldschen-Ohm and Chanda, 2017; Madej and Ziegler, 2018). At the same time, a number of studies have attempted to extend the interpretations from these discrete snapshots into ensemble descriptions reflecting more native environments, such as liposomes rather than detergent micelles. These efforts include cysteine accessibility measurements (Frillingos et al., 1998; Chen and Rudnick, 2000; Javitch et al., 2002; Forster et al., 2006; Zhang and Rudnick, 2006), FRET (Zhao et al., 2010, 2011; Akyuz et al., 2013, 2015; Gregorio et al., 2017), pulsed electron–electron double resonance (PELDOR; also known as double electron–electron resonance [DEER]; Smirnova et al., 2007; Endeward et al., 2009; Zou et al., 2009; Hänelt et al., 2013; Kazmier et al., 2014a,b; Fowler et al., 2015; Timachi et al., 2017), and hydrogen deuterium exchange mass spectrometry (Zhang et al., 2010; Adhikary et al., 2017; Eisinger et al., 2017; Giladi et al., 2017; Reading et al., 2017). In PELDOR, a spectroscopic signal resulting from the interaction between two spin labels in a molecular ensemble is used to derive a probability distribution of the spin-to-spin distance. Typically, a pair of nitroxide radicals is covalently attached to cysteine residues introduced at specific positions in the protein using site-directed spin labeling. A major challenge, however, has been the quantitative interpretation of such biophysical measurements in the context of known structures. Broad signals due to the flexible nature of paramagnetic probes can be exacerbated by the underlying dynamics of a given segment, even for a single state of a protein (McHaourab et al., 2011). The resultant distance distributions may also deviate from the expected values based on x-ray crystal structures due to mismatch between the conditions of the experiment, such as detergent solubilization, temperature, or the presence of a crystal lattice.

In principle, MD simulations provide a means to overcome such challenges, as they can be used to generate ensembles of spin-label configurations for different protein conformations. Unfortunately, however, conventional MD simulations will typically fail to reproduce the probability distributions derived from experiment, due to insufficient computational sampling (i.e., limited simulation time, errors in the force field, or the starting structure used in the simulation is not a major contributor to the experimental data). Thus, advanced simulation strategies have been developed to reproduce the experimental distribution directly, with the minimum perturbation relative to conventional simulations (Roux and Islam, 2013; Islam and Roux, 2015; Marinelli and Faraldo-Gómez, 2015).

Here, we illustrate a strategy using one of these so-called maximum-entropy methods, known as ensemble-biased metadynamics (EBMetaD), to identify the state of a membrane protein most likely to correspond to potentially ambiguous spin-label distance distributions. We apply this technique to the sodium-coupled betaine transporter betaine permease (BetP), which is responsible for osmotic stress detection and response in the soil bacterium *Corynebacterium glutamicum* (Ziegler et al., 2010). Specifically, we assess the compatibility of available BetP structures (Ressl et al., 2009; Perez et al., 2011b, 2012, 2014; Koshy et al., 2013) with PELDOR distance distributions measured under different concentrations of its substrates, sodium and betaine. In this application of EBMetaD, compatibility is measured using the amount of work required for a given input structure to match the experimental distribution (Hustedt et al., 2018), allowing us to rank each simulated structure based on the ease or difficulty of targeting the experimental distribution. This approach provides an analysis of membrane protein conformational ensemble data in a manner that is both fully atomistic and quantitative.

## Materials and methods

### Site-directed mutagenesis

Site-directed mutagenesis was performed with the QuickChange Site-directed Mutagenesis kit II (Stratagen) and PfuUltra DNA polymerase in pASK-IBA5betP plasmid (Schiller et al., 2004). All the plasmids were fully sequenced to confirm the specific mutation.

### Protein expression and purification

Cys-less and double-cysteine variants of BetP were produced and purified as described previously (Rübenhagen et al., 2000) using the primers given in Table S1. Briefly, pASK-IBA5betP WT and mutants were transformed and heterologously expressed in *Escherichia coli* One Shot Invitrogen DH5α-T1. The cells were grown in Lysogeny broth medium with carbenicillin (50 µg/ml) at 37°C. Protein expression was induced with anhydrotetracycline (200 µg/liter). After centrifugation at 4°C, the cells were resuspended in a buffer containing 50 mM Tris-HCl, pH 7.5, 17.2% glycerol, and 1 mM protease inhibitor pefabloc. Membranes were isolated from broken cells by centrifugation and subsequently solubilized with 1% β-dodecyl-maltoside (DDM). Solubilized membranes were loaded on a preequilibrated Strep-Tactin Macroprep (IBA) column and washed with 40 column volumes of 50 mM Tris-HCl, pH 7.5, 200 mM NaCl, 8.6% glycerol, and 0.1% DDM. The protein was eluted in the same buffer supplemented with 5 mM desthiobiotin. All purification steps were performed at 4°C. BetP was further purified to remove the unbound spin label by size-exclusion chromatography (SEC). The protein solution was loaded in a superose 6 10/300 column connected to an Äkta system; the column was preequilibrated with 25 mM Tris-HCl, pH 7.5, 200 mM NaCl, and 0.1% DDM.

### Uptake assays

The protein was reconstituted in *E. coli* polar lipid extract (Avanti) as described (Rigaud et al., 1995; Rübenhagen et al., 2000). Liposomes (20 mg/ml) were prepared by extrusion through a filter (polycarbonate membrane; pore size of 400 nm; Avestin) and diluted 1:4 in 100 mM potassium phosphate (KP_i_), pH 7.5. The solution was titrated with 10% (wt/vol) Triton X-100 and then mixed with the purified protein at a lipid-to-protein ratio of 30:1 for uptake experiments. The detergent was removed by adding BioBeads SM-2 (Bio-Rad) at ratios (wt/wt) of 5 (BioBeads/Triton X-100) and 10 (BioBeads/DDM) in five steps. The proteoliposomes were centrifuged, washed, and resuspended in 100 mM KP_i_, pH 7.5, buffer to a concentration of 60 mg/ml before freezing in liquid nitrogen and storing at −80°C.

Uptake of [^14^C]-labeled glycine betaine was measured as described (Rübenhagen et al., 2000). Briefly, proteoliposomes were extruded (polycarbonate membrane; pore size of 400 nm; Avestin) and centrifuged. Afterward they were resuspended in internal buffer (100 mM KP_i_, pH 7.5) to a lipid concentration of 60 mg/ml. Uptake measurement was initiated by diluting proteoliposomes at a ratio of 1:200 in the external buffer (20 mM NaP_i_, pH 7.5, 25 mM NaCl, 50 µM [^14^C]-labeled glycine betaine, and 1 µM valinomycin). The external osmolarities were adjusted to 0.6 osmol/kg with proline. Samples were filtered for various times through nitrocellulose filters, and the amount of [^14^C]-glycine betaine incorporated into the proteoliposomes during uptake was determined by scintillation counting.

### Site-directed spin labeling

C252T/G450C/S516C-BetP was labeled with a 30-fold molar excess of the spin label (1-oxyl-2,2,5,5-tetramethylpyrrolidin-3-yl)methyl methanethiosulfonate [R5], MTSSL). Free spin label was removed by SEC (see Protein expression and purification). The spin-label concentration was estimated by continuous wave electron paramagnetic resonance (EPR) measurements performed at X-band frequency (9.4 GHz) and used to estimate labeling efficiency based on the protein concentration determined using amido black. Free spin labels were observed at less than 5% in the sample after SEC purification.

### Protein reconstitution for PELDOR measurements

Similar to the protein reconstitution for uptake, the spin-labeled protein was added to extruded liposomes (in 200 mM Tris-HCl, pH 7.5) with a lipid to protein ratio of 20:1 for PELDOR measurements. After incubation, the lipid/protein mixture was transferred to a dialysis membrane (mol wt cutoff, 12–14 kD; Spectrum Laboratories). BioBead SM-2 at ratios (wt/wt) of 5 (BioBeads/Triton X-100) and 10 (BioBeads/DDM) was added to the dialysis buffer in four steps. The proteoliposomes were centrifuged and resuspended in two different buffers for the experimental conditions assessed, prepared in deuterated water; saturating concentrations of sodium (200 mM Tris-HCl, pH 8.0, and 500 mM NaCl) or saturating concentrations of both sodium and betaine (200 mM Tris-HCl, pH 8.0, 300 mM NaCl, and 5 mM betaine). After an additional extrusion and centrifugation step, the proteoliposomes were again resuspended in the corresponding buffers.

### PELDOR measurements

All PELDOR data were recorded on an ELEXSYS E580 EPR spectrometer (Bruker) equipped with a PELDOR unit (E580–400U; Bruker), a Bruker-D2 resonator for Q-Band frequencies using a 10W Amplifier, a continuous-flow helium cryostat (CF935; Oxford Instruments), and a temperature control system (ITC 502; Oxford Instruments). A dead time–free, four-pulse sequence was used with phase-cycled π/2, electron spin-echo envelope modulation averaging (8 × 16 ns), 20 ns pump, and 32 ns detection pulses (Pannier et al., 2000).

### Rotamer library-based predictions of spin-label positions

Although BetP is constitutively a trimer, oligomerization is only required for the response to osmotic stress, which is often referred to as activation (Perez et al., 2011b). The activation process involves the terminal domains (Ott et al., 2008) and modulates (accelerates) the transport kinetics in a manner that depends on intracellular potassium concentration (Rübenhagen et al., 2001) and lipid composition (Schiller et al., 2006). However, activation is not required for the Na^+^-coupled uptake of betaine, which occurs at a basal level in the absence of potassium or osmotic stimulation, and which is retained in monomeric mutants (Perez et al., 2011b). Thus, for the purposes of this analysis, we were interested in the distances only within individual protomers. We therefore used rotamer-library based predictions using Multiscale Modeling of Macromolecules (MMM) 2015.1 (Polyhach and Jeschke, 2010; Polyhach et al., 2011; Jeschke, 2012) to identify the range of possible distances accessible to both inter- and intraprotomer spin–spin distances. The range of distance values between protomers in the trimer was estimated using R1 probes attached to positions G450 and S516 on the periplasmic surface of the inward-open trimer (PDB accession no. 4C7R; Koshy et al., 2013). To predict distance distribution ranges within a protomer, and its dependence on the conformation of the protein, spin-label rotamers were modeled onto structures of the outward-open (chain A of PDB accession no. 4LLH; Perez et al., 2014), or inward-open (chain A of PDB accession no. 4C7R) states. Since the standard spin-label rotamer libraries (R1A_175K and R1A_298K) included, in some cases, only a few rotamers, we used the R1A_298K_xray library (Polyhach and Jeschke, 2010; Polyhach et al., 2011; Jeschke, 2012).

**Figure 1.**
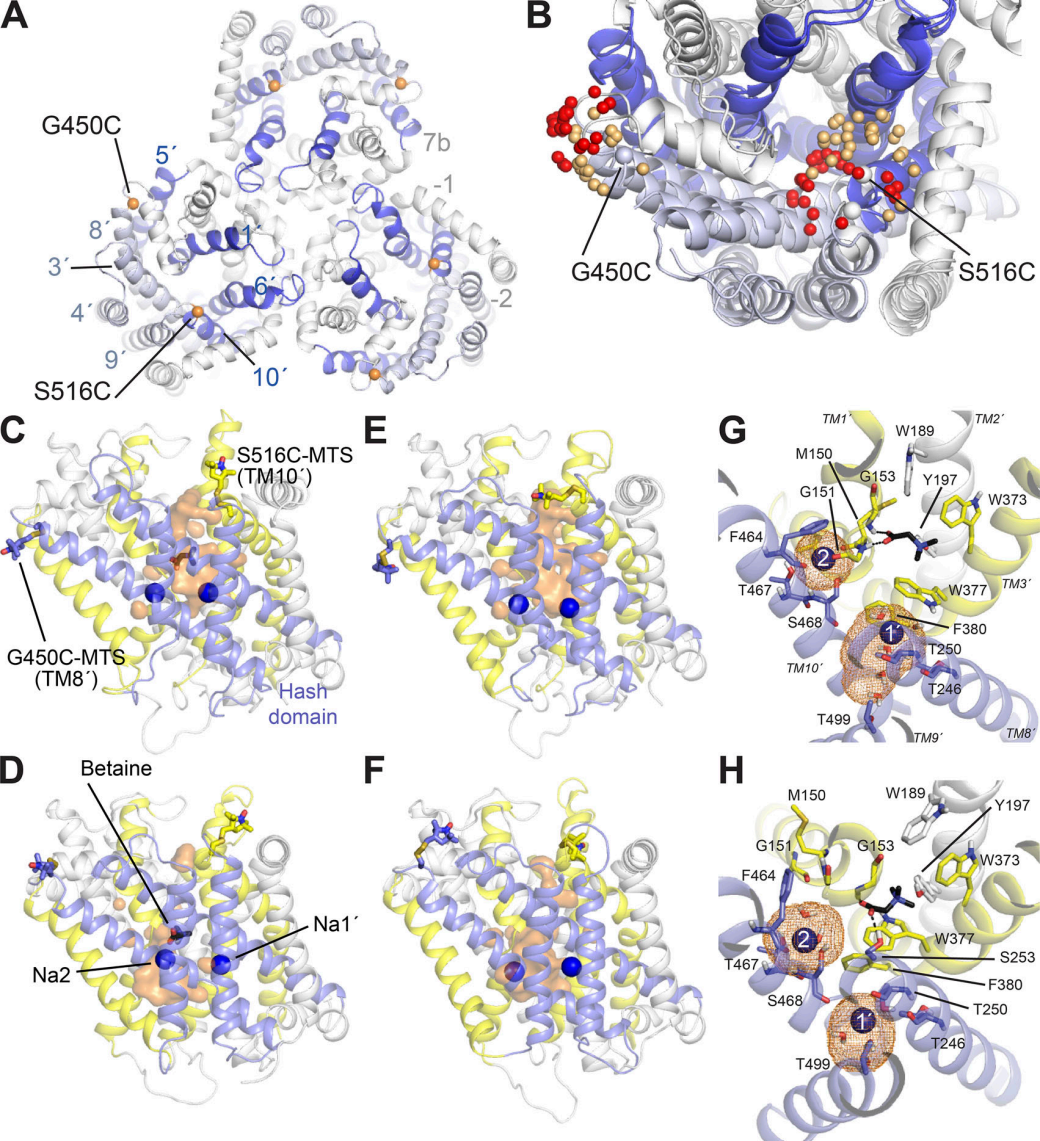
**Structure of BetP and spin-label locations. (A and B)** The structure of BetP shown as cartoon helices, highlighting TM helices 1′, 5′, 6′, and 8′ lining the substrate pathway (blue) and helices 3′, 4′, 8′, and 9′ in the hash domain (pale blue). Helices −2, −1, and 7b (white) contribute to the trimer interface. Each BetP protein chain was labeled at two cysteines on the periplasmic surface, introduced at positions G450 and S516. (A) Trimer structure of BetP (PDB accession no. 4C7R). Cα atoms of labeled positions are shown as orange spheres. (B) Comparison of MMM-based predicted spin-label positions in structures of the two extreme conformations of monomeric BetP, outward-open (PDB accession no. 4LLH, chain A) or inward-open (PDB accession no. 4C7R, chain A), with nitroxide N atom positions colored red and gold, respectively. **(C–F)** Simulation system for spin-labeled monomeric BetP. Snapshots indicate the location of the spin labels with betaine bound (C and D) or without betaine (E and F), in either outward-open (C and E) or inward-open (D and F) conformations. The protein is viewed from within the plane of the membrane. The MTS labels and bound betaine are shown as sticks, and sodium ions are shown as blue spheres. Representative snapshots were selected from the center of a cluster of conformations in which the probe distance is at the peak of the distribution (∼42 Å or ∼27 Å for the betaine-bound or betaine-free systems, respectively). Water molecules within 10 Å of the ligand are shown as an orange surface, highlighting the pathways on the extracellular or intracellular sides. Helices 3′, 4′, 8′ (including G450C), and 9′, which comprise the so-called hash domain, are colored blue. Helices 1′, 5′, 6′, and 10′ (including S516C) are colored yellow. Lipids and surrounding solvent are omitted for clarity. **(G and H)** Sodium ion binding sites (Na1′ and Na2, labeled 1′ and 2, respectively) in the outward- (G) and inward-open (H) conformations of BetP in the presence of substrate, viewed from the extracellular side. The density occupied by each ion during the EBMetaD simulations is shown as orange mesh. Nearby water molecules are shown in stick representation.

Another consequence of BetP being a trimer is that the six attached labels may produce ghost signals due to multispin effects, which could mask peaks or create additional peaks at short distances (Jeschke et al., 2009; von Hagens et al., 2013). We therefore took steps during data processing to mitigate such effects (see Supplemental text). In addition, rotamer library–based predictions suggest that these effects will be negligible in this case (see Fig. 1, A and B; and Supplemental text).

### MD simulation setup: Protein preparation

Simulations were set up for monomers of BetP isolated from three different trimeric crystal structures. These structures represent the substrate-free inward conformation (PDB accession no. 4C7R chain C, 2.7 Å resolution; Koshy et al., 2013), the substrate-bound inward-open state (PDB accession no. 4AIN chain C, 3.1 Å resolution; Perez et al., 2012), and outward-open, substrate-free and substrate-bound states (PDB accession no. 4LLH chain A, 2.95 Å resolution; Perez et al., 2014).

The structure of the outward-open conformation of BetP (4LLH) was determined using a choline-specific version of BetP carrying a G153D point mutation (Perez et al., 2014), which we mutated back to the WT sequence (D153G). A betaine molecule was positioned at the choline binding site for the substrate-bound simulation of D153G by superposing the trimethylamine groups (Fig. 1 G), thereby recapitulating betaine–protein interactions observed in the fully occluded state. We considered the possibility that the system would shift away from the outward-facing conformation due to the replacement of the substrate, and the elimination of a bulkier ionizable group at position 153. To analyze the stability of the system, therefore, we computed the number of waters present in the outer cavity (defined as a box bounded by the Cα atoms of residues Met150 and Ile375 in x, residues Met150 and Thr250 in y, and Met150 and Ser365 in z) during 100 ns of unrestrained MD simulation. The pathway maintained a constant hydration of 28.3 ± 4.1 or 30.0 ± 3.5 waters for the substrate-bound and -free systems, respectively, indicating that, at least on the simulated timescales, the reverse D153G substitution did not adversely destabilize the outward-open conformation.

Histidine residues were protonated as described previously (Perez et al., 2014). Glu161 was modeled as neutral, based on the strong shift in logarithm of the acid dissociation constant, p*K*_a_, predicted for outward-facing, fully-occluded, and inward-facing x-ray structures of BetP using Multi-Conformation Continuum Electrostatics (MCCE) with the protein dielectric constant set to either 4 or 8 (Alexov and Gunner, 1997). Using MCCE, Asp239 was predicted to be deprotonated, but visualization of the contacts involving Asp239 during molecular simulations suggested this configuration was unstable. To quantify this, we tracked 36 pairs of interatomic distances (between the Cγ and N atoms of Asp239; Oγ and N of Ser218; O of Gly235; N of Leu217; O of Leu237; and O of Leu241) during 100-ns-long unrestrained simulations of the outward-open, substrate-bound structure, to assess whether the distances differed significantly from those in the reference x-ray structure. The number of pairs whose distances were considered inconsistent with the x-ray structure (i.e., for which the average distance over the simulation is >2 SDs from the reference) was substantially smaller for the protonated (8.3 ± 0.3) than the deprotonated form of Asp239 (30.5 ± 2.8). Thus, Asp239 was treated as protonated in the EBmetaD calculations. All other ionizable residues were set to their physiological protonation states, including Asp470, whose predicted p*K*_a_ in outward-facing conformations was close to its physiological value (∼4). The p*K*_a_ of Asp470 was shifted higher (∼7) in other conformations depending on the occupancy of the Na2 site. Since all our simulations include a sodium ion at Na2, we focus on results obtained with Asp470 deprotonated. However, all simulations were repeated with Asp470 protonated.

### MD simulation equilibration

All proteins were embedded in a hydrated (∼15,000 waters) bilayer of 219 palmitoyl oleyl phosphatidyl-glycerol lipids with GRId-based Force Field INput (GRIFFIN; Staritzbichler et al., 2011). All systems were set to neutral by modifying the number of sodium (254–259) and chloride ions (25–30) in the bulk water.

Each system was subsequently equilibrated for 12 ns through a series of restrained simulations, involving 2-ns trajectories with stepwise release of harmonic positional restraints, as follows: (a) 15 kcal/mol Å^−2^ applied to side chains, backbone, and ligands; (b) backbone and ligand restraints of 15 kcal/mol Å^−2^ and side chain restraints of 4 kcal/mol Å^−2^; (c) Cα atom and ligand restraints of 15 kcal/mol Å^−2^, backbone restraints of 4 kcal/mol Å^−2^, and side chain restraints of 1 kcal/mol Å^−2^; and (d) ligand restraints of 15 kcal/mol Å^−2^, Cα atom restraints of 4 kcal/mol A^−2^, and side chain restraints of 1 kcal/mol Å^−2^. In the final, 4-ns-long step, the Cα atom restraint was 1 kcal/mol Å^−2^, and the ligand restraint was 4 kcal/mol Å^−2^. This was followed by 108 ns of unconstrained equilibration. Pairs of R5 spin labels with the R stereoisomer of the chiral center were added at Gly450 and Ser516 (Fig. 1, C–F) using the Chemistry at HARvard Macromolecular Mechanics graphical-user interface (CHARMM-GUI; Jo et al., 2008), and the labeled system was further equilibrated for 100 ns.

### MD simulation: Ion binding sites

The effect of substrates on the distance distributions of the probes was measured with both sodium and betaine, and compared with a substrate-free condition in which only sodium was present. For comparison with the latter condition, we simulated BetP without betaine bound, but with sodium ions placed at both Na1′ and Na2 binding sites (Khafizov et al., 2012). Structural evidence for the modeled Na2 site is unambiguous, with densities observed in both fully occluded (4AIN chain B) and outward-open (4LLH chain A) structures of BetP (Perez et al., 2012, 2014). On the other hand, the Na1′ site ion has not been detected structurally, even in the fully occluded conformation, despite the saturating sodium concentrations in the crystallization buffer (>100 mM Na^+^, compared with a *K*_d_ for sodium of ∼53 mM; Khafizov et al., 2012). Nevertheless, strong evidence for the Na1′ site has been obtained from an array of biochemical, biophysical, and simulation data (Khafizov et al., 2012). For any given structure or site therein, the lack of a clear density for a sodium ion may reflect the moderate resolution of the structural data, or the possibility that the effective affinity of that state for sodium is lower than the measured overall *K*_d_, which reflects an ensemble of protein conformations. For consistency across simulation setups, therefore, we restrained the substrates loosely to their binding sites (Fig. 1, G and H). This prevents ions or betaine from unbinding from the simulated open structures, while not explicitly defining their exact (unknown) coordination, or causing local distortions in the protein. Specifically, we apply restraints based on a coordination number (*C_num_*) variable, defined as

Cnum=∑j1−(rijR)81−(rijR)10, (1)

where the *i* index refers to the ligand (sodium ion or betaine nitrogen atom), *j* indicates the protein atoms coordinating this ligand, and *r_ij_* is the distance between these atoms. The Na1′ site was defined as the side chain oxygen of residues Thr250 and Thr246 and the backbone oxygen of Thr246 (Khafizov et al., 2012). The Na2 site was defined as the oxygen of the side chains of Thr467 and Ser468 and the backbone side chain of Met150. The betaine nitrogen atom was coordinated with the Cγ atom of Trp377, the Oη atom of Tyr197, and the backbone oxygen of Ala148. The cutoff, *R*, was set to 2.5 Å for sodium ion coordination and to 2.0 Å for betaine coordination. A harmonic restraint of 50 kcal/mol was applied that limits the coordination to *C_num_*(Na1′) ≥ 1.3 contacts, *C_num_*(Na2) ≥ 2.1 contacts, and *C_num_*(betaine) ≥ 2.0 contacts.

### MD simulation parameters

All simulations were performed using NAMD v2.9 (Phillips et al., 2005). The CHARMM36 force field (Klauda et al., 2010; Best et al., 2012; Venable et al., 2015) was used for the protein, lipid, and ions; TIP3P for the water molecules (Jorgensen et al., 1983); and betaine parameters from Ma et al. (2010). The simulations were performed at constant temperature (298°K) and pressure (1 atm), imposed with a Nosé-Hoover Langevin barostat and thermostat. The membrane area was kept constant, and periodic boundary conditions were used in all directions. The simulation time step was 2 fs. Electrostatic interactions were calculated using particle mesh Ewald with a real-space cutoff of 12 Å. The cutoff for van der Waals interactions was also set to 12 Å. A switching function starting at 10 Å was applied to both electrostatic and van der Waals interactions.

### Biasing the simulated ensemble to the experimental distance distribution

The collective variables module (Fiorin et al., 2013) was used to apply the ligand coordination number–based constraints. EBMetaD calculations (Marinelli and Faraldo-Gómez, 2015) were performed with a modified version of PLUMED v1.3 (Bonomi et al., 2009) provided by F. Marinelli and J.D. Faraldo-Gómez (Theoretical Molecular Biophysics Section, National Heart, Lung, and Blood Institute, National Institutes of Health, Bethesda, MD). The input syntax is provided in the Supplemental EBMetaD command file. This code has since been implemented in NAMD v2.13 within the collective variables module, and the corresponding input commands are also provided as a Supplemental text file. The target distance distribution was enforced on the centers of mass of the two spin-label nitroxide bonds. Briefly, EBMetaD progressively adds adaptive biasing potentials in the form of additive Gaussians to the underlying force field during the simulations. The biasing potentials were deposited every 2 ps, and the width of the Gaussians was 0.5 Å. Unlike canonical metadynamics, the height of each applied Gaussian depends on the probability of the targeted distribution, such that the Gaussian will be larger for low probability regions and smaller for high probability regions. Importantly, the bias introduced during the simulations is the minimum necessary to fulfill the target distribution, preventing overfitting to the experimental distance data.

### Analysis of the unbiased trajectories

Structural similarity was measured as the RMSD in the position of the backbone atoms, after fitting on the same atoms. The segments lining the extracellular pathway were defined as the periplasmic half of transmembrane (TM) segments 1′, 3′, 4′, 6′, 8′, 9′, and 10′ (residues 152–167, 251–265, 277–286, 359–373, 450–462, 500–510, and 515–525) and the cytoplasmic pathway using the cytoplasmic half of TM segments 1′, 3′, 4′, 5′, 6′, 8′, and 9′ (residues 137–151, 235–250, 287–294, 301–314, 374–388, 463–479, and 488–499).

### Analysis of the applied work and biased trajectories

From the bias potential applied during a simulation, we define the work along the distance distribution, *W*, to be

$$W=kT\;ln\int^{\text{​}}\rho (r)e^{\left(\frac{\bar{V}\left(r\right)-\bar{V}}{kT}\right)}dr,$$ (2)

where *r* is the nitroxide–nitroxide distance, *ρ(r)* is the experimental distance distribution being targeted, *k* is the Boltzmann constant, and *T* is the temperature. The quantity

$$\bar{V}\left(r\right)={\left(t_{tot}-t_{f}\right)}^{-1}\overset{t_{tot}}{\underset{t_{f}}{\int }}V\left(r,t\right)dt$$

is the average bias potential added during a simulation of length *t_tot_*, in which V(r,t) is the bias potential added after *t* timesteps. Here, $\bar{V}\left(r\right)$ is averaged over the last 0.8 µs of each 1-µs-long enhanced simulation, i.e., excluding the initial “filling” time, $t_{f}=$ 0.2 µs. The offset term $\bar{V}=\int^{\text{​}}\rho (r)\bar{V}\left(r\right)dr$ is defined as the mean value of $\bar{V}\left(r\right)$ over the experimental distance distribution and allows for comparison between simulations with different starting structures.

Note that the work can also be written as

$$W=kT\;D_{KL},$$ (3)

where *D_KL_* is the Kullback-Leibler divergence between biased and debiased distributions (Eq. 30 in Hustedt et al., 2018).

To evaluate the effect of the bias on specific features of the simulations, debiased distributions were computed as follows:

$$\rho {\left(r\right)}_{debiased}=\frac{e^{\frac{-F\left(r\right)}{kT}}}{C},$$ (4)

where $F\left(r\right)=-\bar{V}\left(r\right)-kTln\left(\rho \left(r\right)\right)$ is the free energy of an unbiased simulation as a function of the interspin distance, r (Eq. 6 in Marinelli and Faraldo-Gómez, 2015), and *C* is a normalization factor,

$$C=\int^{\text{​}}e^{\frac{-F\left(r\right)}{kT}}dr.$$

To isolate distances potentially arising from interprotomer coupling, we computed the work required to bias the trajectory to the distribution only in a specific distance range. To this end, in the work calculation we considered a modified average bias potential, $\bar{V}{\left(r\right)}_{r<r_{\max}},$ which was assumed to be flat for distances above a threshold *r*_max_, which was set to 37 Å. In this case, we also modified the reference experimental distribution ($\rho_{modified}$) to account for the bias potential change. Thus,

$$\rho {\left(r\right)}_{modified}=\frac{e^{\frac{-F\left(r\right)-\bar{V}{\left(r\right)}_{r<r_{\max}}}{kT}}}{C^{'}},$$ (5)

where *C*´ is a normalization factor,

$$C'=\int^{\text{​}}e^{\frac{-F\left(r\right)-\bar{V}{\left(r\right)}_{r<r_{\max}}}{kT}}dr.$$

### Online supplemental material

Supplemental text describes the processing and analysis of multi-spin dipolar contributions, along with EBmetaD input commands for NAMD. Fig. S1 presents simulated PELDOR data examining the likelihood of ghost peaks due to multi-spin effects. Fig. S2 presents spin-label distance distributions predicted by sampling spin-label rotamers on static crystal structures, and filtering of long-range peaks. Table S1 presents primer sequences. Table S2 presents reference distances in x-ray structures of BetP.

## Results

### Site-directed spin labeling of BetP

**Figure 2.**
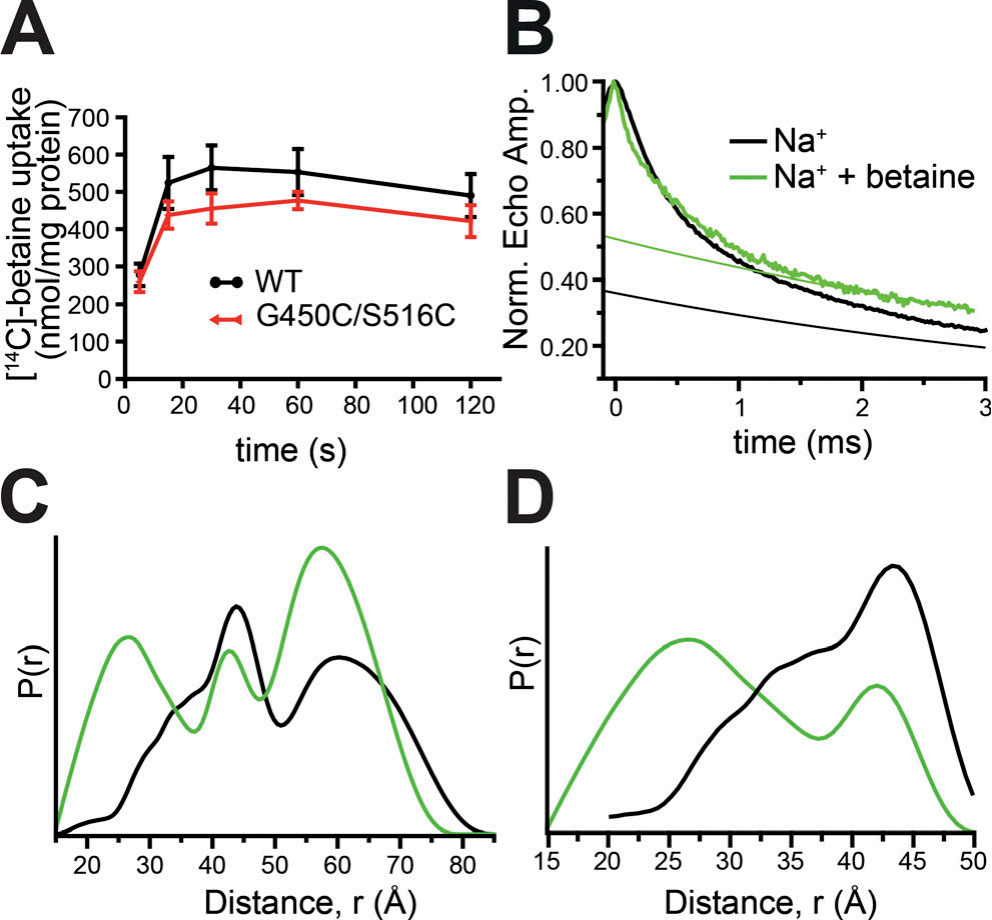
**Measurements for BetP in *E. coli* polar lipid proteoliposomes with spin labels introduced at positions 450 and 516. (A)** Uptake of betaine in nanomolar per milligram protein was measured at 0.6 osmol/kg as a function of time for WT and MTSSL-labeled BetP cysteine variant C252T/G450C/S516C reconstituted into *E. coli* polar lipid liposomes. Uptake was initiated by adding saturating concentrations of 50 µM [^14^C]-betaine. Each value is the mean ± SEM of at least six independent measurements. **(B)** PELDOR normalized echo amplitude (Norm. Echo Amp.) time traces and assumed background traces (thinner smooth lines) for MTSSL-labeled BetP measured in the presence of 500 mM NaCl (black) or 300 mM NaCl and 5 mM betaine (green). **(C and D)** Probability of a distance *P*(*r*) versus distance (*r*) between spins derived from Tikonov regularization of the PELDOR time traces in B, before (C) and after (D) subtraction of a Gaussian distribution with a peak at ∼60 Å (see Fig. S2).

Nitroxide radicals were introduced into the cysteine-less BetP mutant C252T (Rübenhagen et al., 2000; Ott et al., 2008) by site-directed spin labeling. BetP is a homotrimer (Fig. 1 A), but each protomer is functional for sodium-coupled betaine transport and operates independently (Perez et al., 2011a). Therefore, two spin labels must be introduced on each protomer to report on transport-related conformational changes. Here, we use data for BetP labeled at positions either side of the extracellular pathway. The first probe was attached to the flexible TM segment 10′ (S516C), which folds over the extracellular pathway in inward-facing conformations (Fig. 1 B–F; Perez et al., 2012). The second probe is on the periphery at the tip of TM 8′ (G450C; Fig. 1 B). MTS spin labels were covalently linked to these residues with excellent efficiency (112 ± 22.4%), and the labeled construct was capable of sodium-dependent [^14^C]-betaine uptake when reconstituted into liposomes (Fig. 2 A).

### Comparison of spin-label distance distributions for BetP with x-ray crystallographic data

We measured the time dependence of spin–spin interactions between probes attached to G450C/S516C of BetP using PELDOR both in the presence and absence of betaine (Fig. 2 B). The resultant traces were translated into distance distributions (see Materials and methods), revealing multiple peaks that span a very wide distance range, from 20 to 80 Å (Fig. 2 C). Using simulated data and rotamer library spin-label predictions, we ruled out the possibility that one or more of these peaks reflects ghost signals due to multi-spin effects (Supplemental text and Fig. S1). Longer-range peaks in these distance distributions are likely to result from interactions across protomers (Fig. S2). We estimated the extent of these interactions using a rotamer library, and then removed the corresponding peaks from the underlying data by subtracting a Gaussian distribution centered at the corresponding peak (∼60 Å; Fig. 2 D; and Fig. S2, C and D).

Comparison of the distance distributions obtained under the two measured conditions indicates the appearance of a significant population at ∼25 Å upon the addition of betaine (Fig. 2, C and D). An intuitive interpretation of the appearance of this peak might be that, in the presence of all required substrates, the extracellular pathway is able to close, enabling the spins to get closer together. This interpretation would be consistent with the observation that the backbone Cα atoms in the inward-facing structures of BetP are somewhat closer (∼1–2 Å) than in the outward-facing conformation for the equivalent ligand-bound configuration (Table S2). However, the difference in the distances between the crystal structures (Table S2) is substantially smaller than the width of the measured spin-label distance distributions. Such a discrepancy may be accredited in part to the flexibility of the probes, which can lead to peak widths of ∼10 Å even when attached to a rigid protein backbone (Polyhach et al., 2011; Roux and Islam, 2013; Marinelli and Faraldo-Gómez, 2015). However, the protein segment to which the probe(s) are attached may also be more dynamic in one conformation than another. Thus, interpretation of the differences between the distributions in the two conditions is nontrivial.

### MD simulations of BetP inward- and outward-facing states converge on the distance distribution

**Figure 3.**
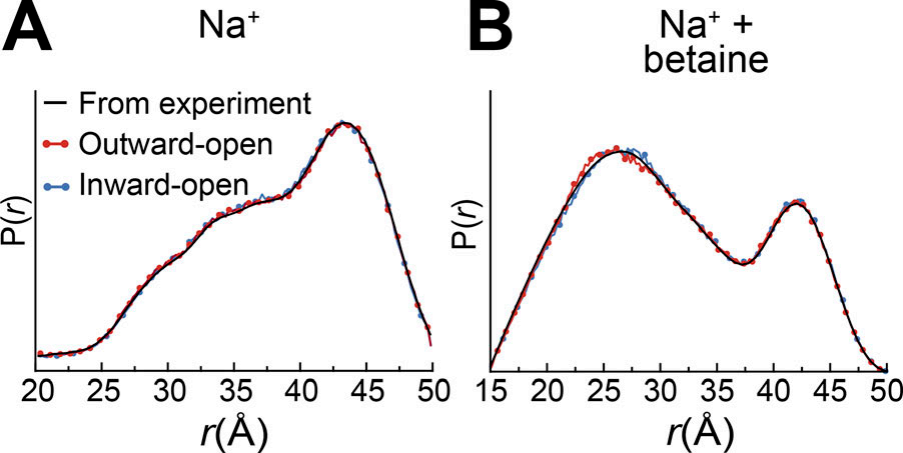
**Convergence of simulated to experimental distance distributions.** The probability of a distance *P*(*r*) is plotted versus distance (*r*). The PELDOR-based distances (black lines), measured in the presence of 500 mM NaCl (A) or 300 mM NaCl plus 5 mM betaine (B), are compared with distances obtained in 1-µs-long EBMetaD MD simulations, performed for BetP monomers in the presence of two sodium ions (A) or with two sodium ions plus a betaine substrate (B). The simulations were started with BetP structures of either outward-facing (PDB accession no. 4LLH chain A, red circles) or inward-facing (PDB accession no. 4C7R, blue circles) conformations.

**Figure 4.**
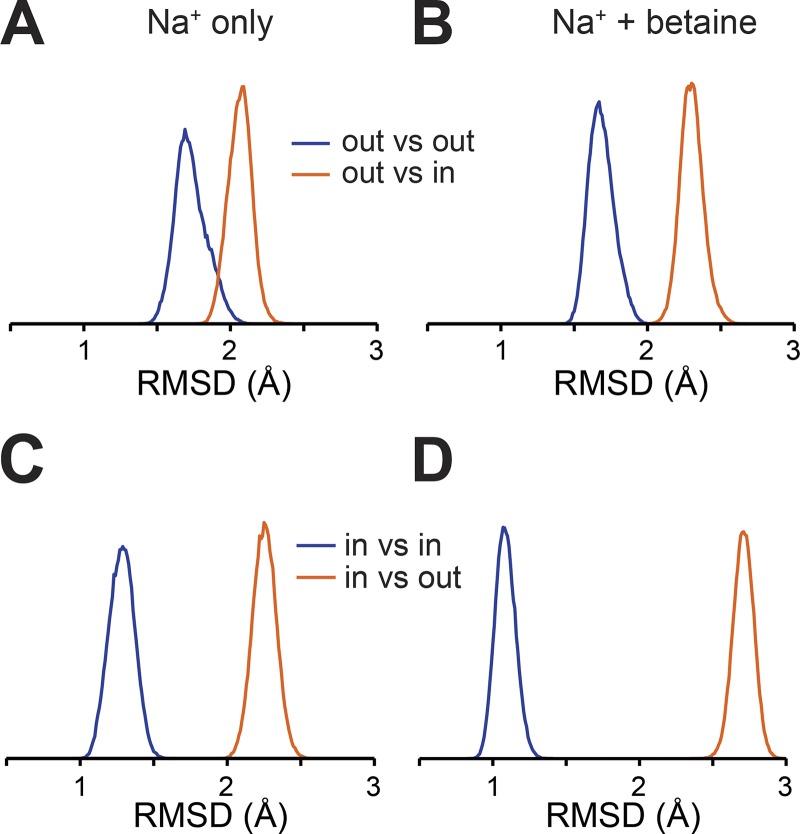
**The EBMetaD bias does not change the overall conformation of the protein. (A–D)** Structural similarity (in RMSD) of each simulated ensemble with respect to the two extreme conformations of BetP. EBMetaD simulation trajectories initiated with either outward-facing (A and B) or inward-facing (C and D) conformation in the presence of two sodium (A and C) or two sodium and betaine (B and D) are compared with either the initial structure (blue) or with the structure of the opposite state (orange).

Here, we ask which of the known structures of BetP is most consistent with the distances identified for these probes under each of the two conditions. We set up simulation systems of the BetP monomer starting in either outward- or inward-facing conformations, either in the presence of two Na^+^ ions alone, or with both ions and betaine (see Materials and methods). The MTS spin label was modeled explicitly at positions 450 and 415, and the labeled protein was embedded in a hydrated lipid bilayer. EBMetaD simulations initiated with each of the two protein conformations and with either of the two distance distributions all converged within 1 µs, even when two peaks were present in the distribution (Fig. 3). This convergence was achieved without a major transition of the structure to the other states; indeed, the conformations of the protein remain close to the input structure, both in the presence and absence of betaine, as measured using the RMSD of the core TM segments (Fig. 4).

### Ranking structures according to the computed work

**Figure 5.**
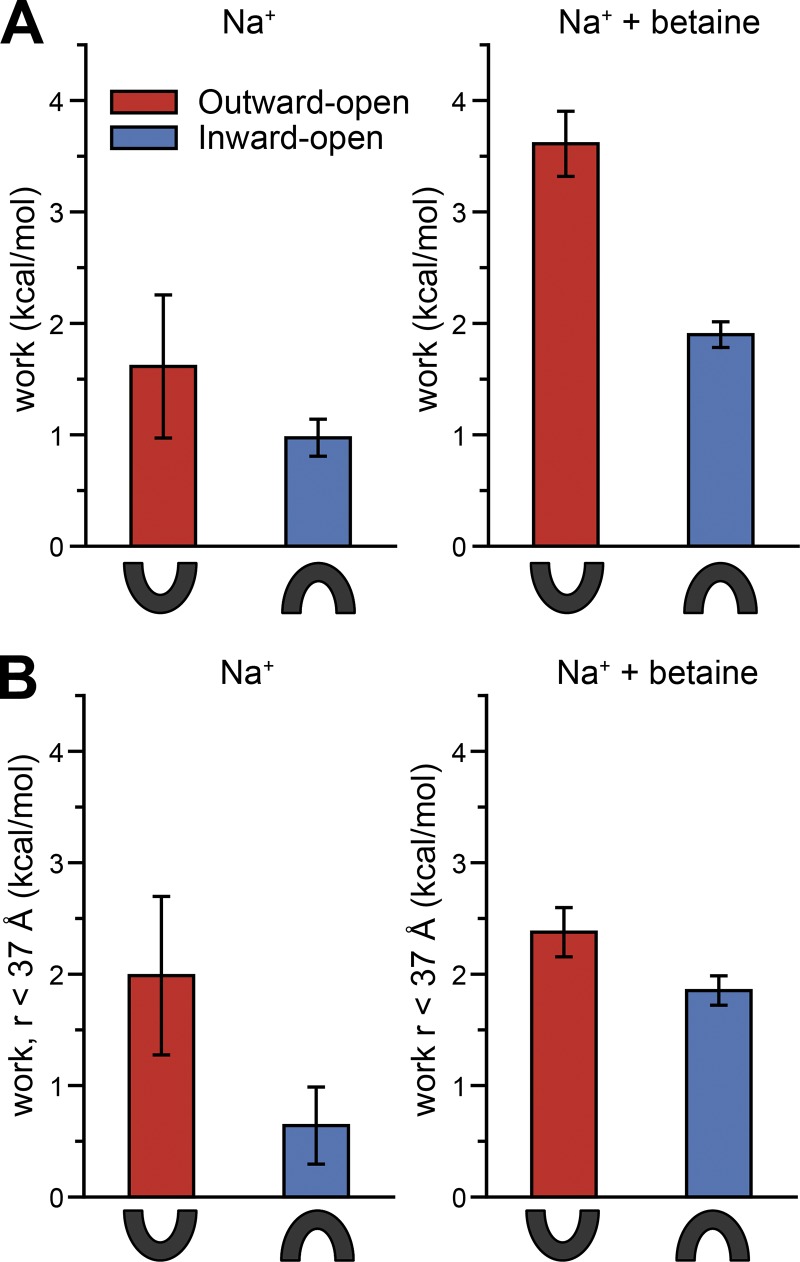
**Work required by each simulation system to reproduce the experimental distance distribution.** The work was computed by averaging the bias potential applied over the last 0.8 µs of each simulation of G450R5/S516R5. Simulations were performed with two bound sodium ions (2Na^+^), in either the absence (left) or presence (right) of betaine. The BetP conformation used to start the simulation was either inward- (PDB accession no. 4C7R, blue) or outward-facing (PDB accession no. 4LLH chain A, red). Error bars represent the SD of work values obtained by averaging over the two halves of the last 0.8 µs for the entire width of the distance distribution (A), or averaging the bias potential applied for distances <37 Å (B; see Materials and methods and Fig. S2), as expected for exclusively interprotomer interactions.

A powerful aspect of the EBmetaD method is that one can track the amount of work that was required to match the distance distribution for any given input molecular system (Hustedt et al., 2018). In the case of BetP, we can therefore ask which of the two conformational states requires the least work to match the distribution, and is thus inherently more consistent with the experimental ensemble. In the presence of sodium alone, matching the spin-label distance distribution starting with the Na^+^-bound inward-facing conformation of BetP required less work than when starting with the Na^+^-bound outward-facing conformation (Fig. 5 A, left), although the effect size is small compared with the error observed for the latter.

A similar trend is observed for the simulations of the substrate-bound state targeted to the experimental distribution measured in the presence of sodium and betaine (Fig. 5 A, right). That is, the inward-facing state required substantially less work to match the distribution than the outward-facing state. Nevertheless, the amount of work in both cases was higher than for the simulations in the absence of betaine, suggesting that the bimodal distribution reflects a mixture of these two states, or possibly a third state not considered explicitly here.

### Sensitivity to contributions at long ranges

A structural interpretation of the above results runs somewhat counter to the intuitive expectation that in the presence of sodium, the extracellular pathway would be open (Fig. 1 B), and that the presence of betaine enables the pathway to close to adopt the inward-facing state. Instead, the above results could be interpreted to suggest that the inward-facing conformation is preferred in the presence of sodium, and thus that the extracellular pathway is closed.

One concern is that, even after parsing out longer-range contributions (Fig. 2, C and D), the distance distribution at short ranges may still contain residual information from interprotomer interactions. To test the sensitivity of the EBmetaD simulations to the contributions at longer distances, we reanalyzed the trajectories by computing the work done in a range that excludes the second peak at ∼42 Å (Fig. S2). The major difference between the targeted distributions is therefore the peak at ∼27 Å that appears in the presence of betaine. The ranking based on this analysis was unchanged (Fig. 5). That is, for both conditions, the inward-facing conformation requires less work to match the distribution at shorter distances (Fig. 5 B). Moreover, in the presence of betaine, both inward- and outward-facing conformations required substantial work (Fig. 5 B), similar to the findings for the entire distribution (Fig. 5 A).

### Sensitivity to other factors in the system

**Figure 6.**
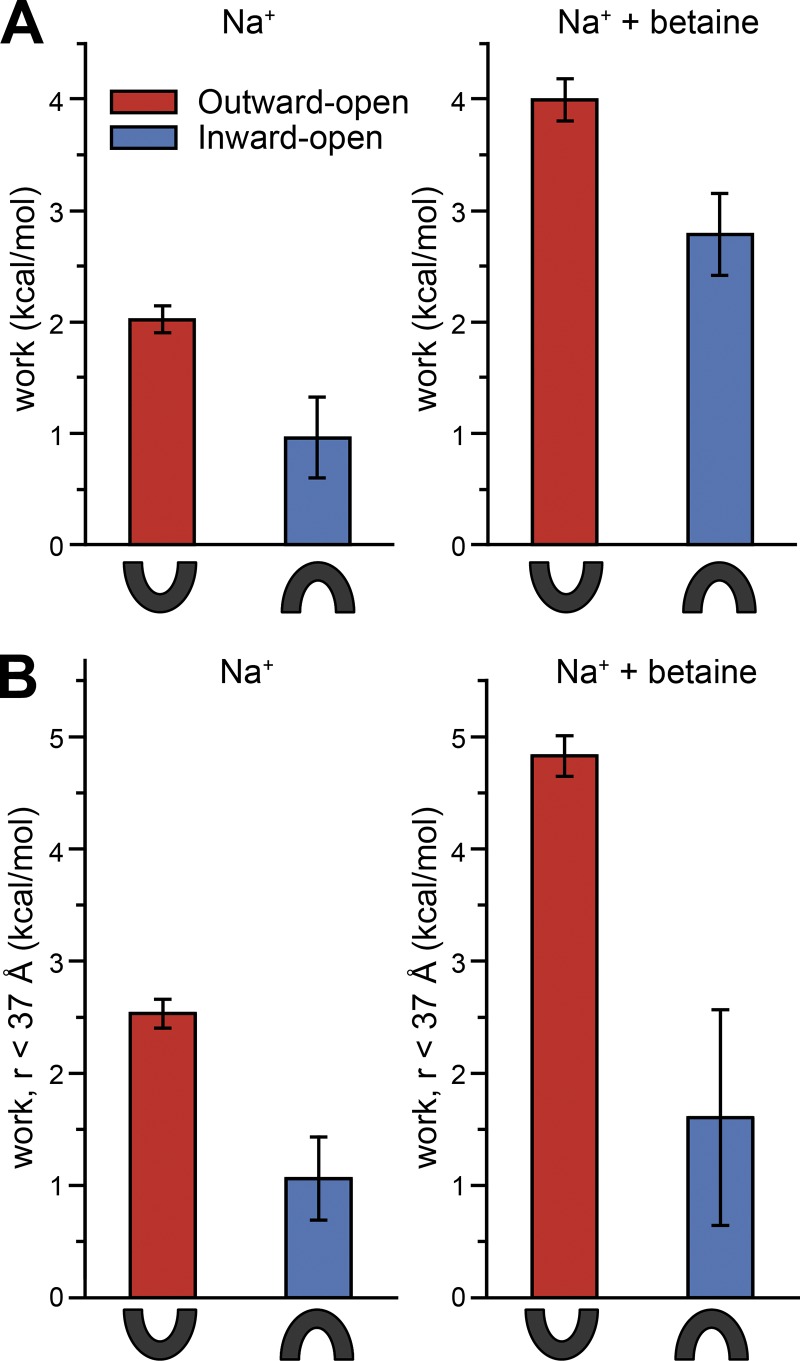
**Work required by each D470-protonated simulation system to reproduce the experimental distance distribution.** The work was computed by averaging the bias potential applied over the last 0.8 µs of each simulation of G450R5/S516R5 with Asp470 protonated. Simulations were performed with two bound sodium ions (2Na^+^), in either the absence (left) or presence (right) of betaine. The BetP conformation used to start the simulation was either inward- (PDB accession no. 4C7R, blue) or outward-facing (PDB accession no. 4LLH chain A, red). Error bars represent the SD of work values obtained by averaging over the two halves of the last 0.8 µs for the entire width of the distance distribution (A), or averaging the bias potential applied for distances <37 Å (B; see Materials and methods), as expected for exclusively inter-protomer interactions.

In the above calculations, the ionizable side chain of Asp470, which is close to the cytoplasmic pathway, was set to be deprotonated, consistent with the low p*K*_a_ predicted for most conformations of the protein (see Materials and methods). However, there are some states for which the p*K*_a_ of Asp470 was predicted to be shifted to higher values (∼7), and even though Asp470 is on the opposite side of the protein from the probes, it is possible that the ranking of states might reflect an energetically unfavorable conformation for BetP when Asp470 is deprotonated. As another test of the sensitivity of the system to the input structure, we therefore performed EBMetaD simulations on all four states of BetP with Asp470 protonated. The work done for all four states allowed the same trend as for the deprotonated forms of BetP (Fig. 6), namely with the inward-facing conformation apparently most compatible with the data obtained in the presence of sodium, while the distance distribution measured with betaine is not readily matched by either one of the two states.

### Molecular interpretation of the work

**Figure 7.**
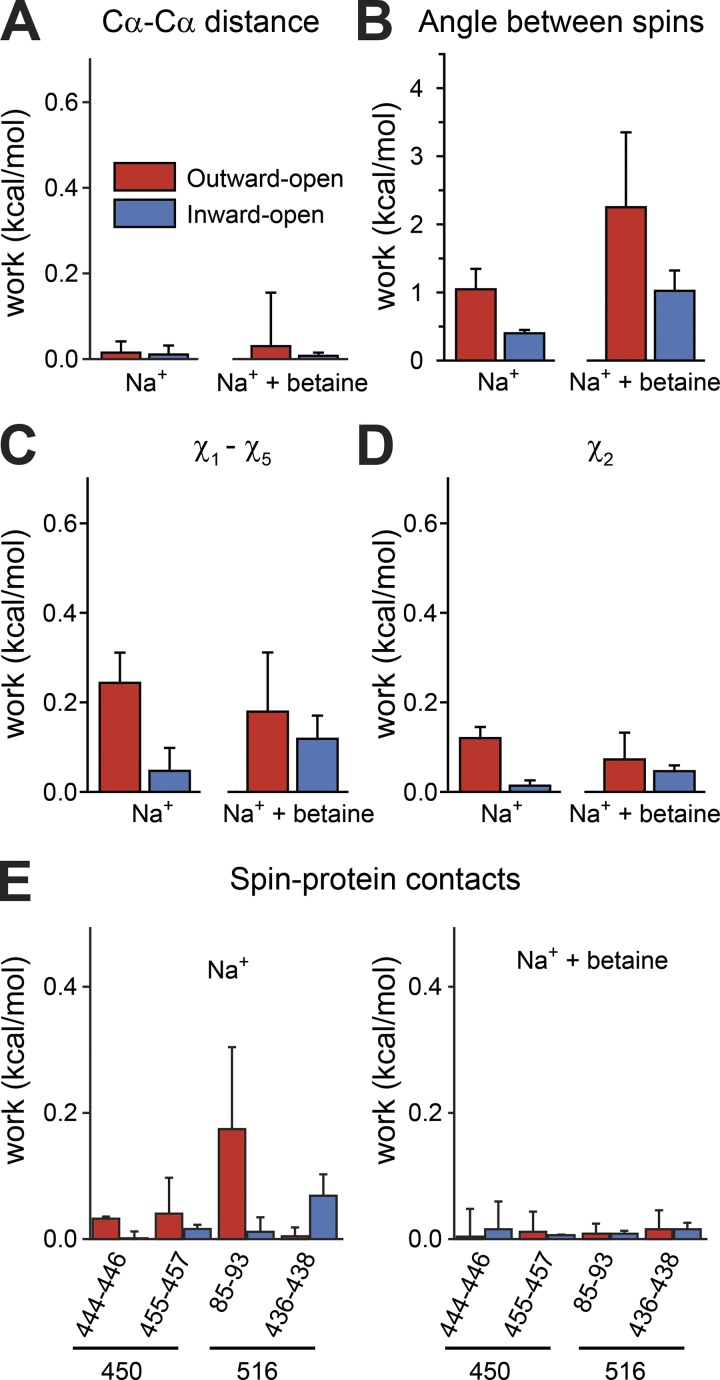
**Breakdown of contributions to the work for each molecular simulation system.** Work computed with the Kullback-Leibler divergence between the biased and debiased trajectories (see Materials and methods) for the distance between the Cα atoms of residues 450 and 516 (A); the relative orientation of the spin labels (B); the side-chain dihedral angles χ_1_ to χ_5_ (C) or only χ_2_ (D); and interactions between the probes at positions 450 and 516 and nearby residues in loop EL5 (residues 436–438 or 444–446), TM8′ (455–457), or TM-1′ (85–93; E). Simulations were performed with two bound sodium ions (Na^+^), in either the absence (left) or presence (right) of betaine. The initial protein conformation was either outward- (PDB accession no. 4LLH chain A, red) or inward-facing (PDB accession no. 4C7R, blue).

**Figure 8.**
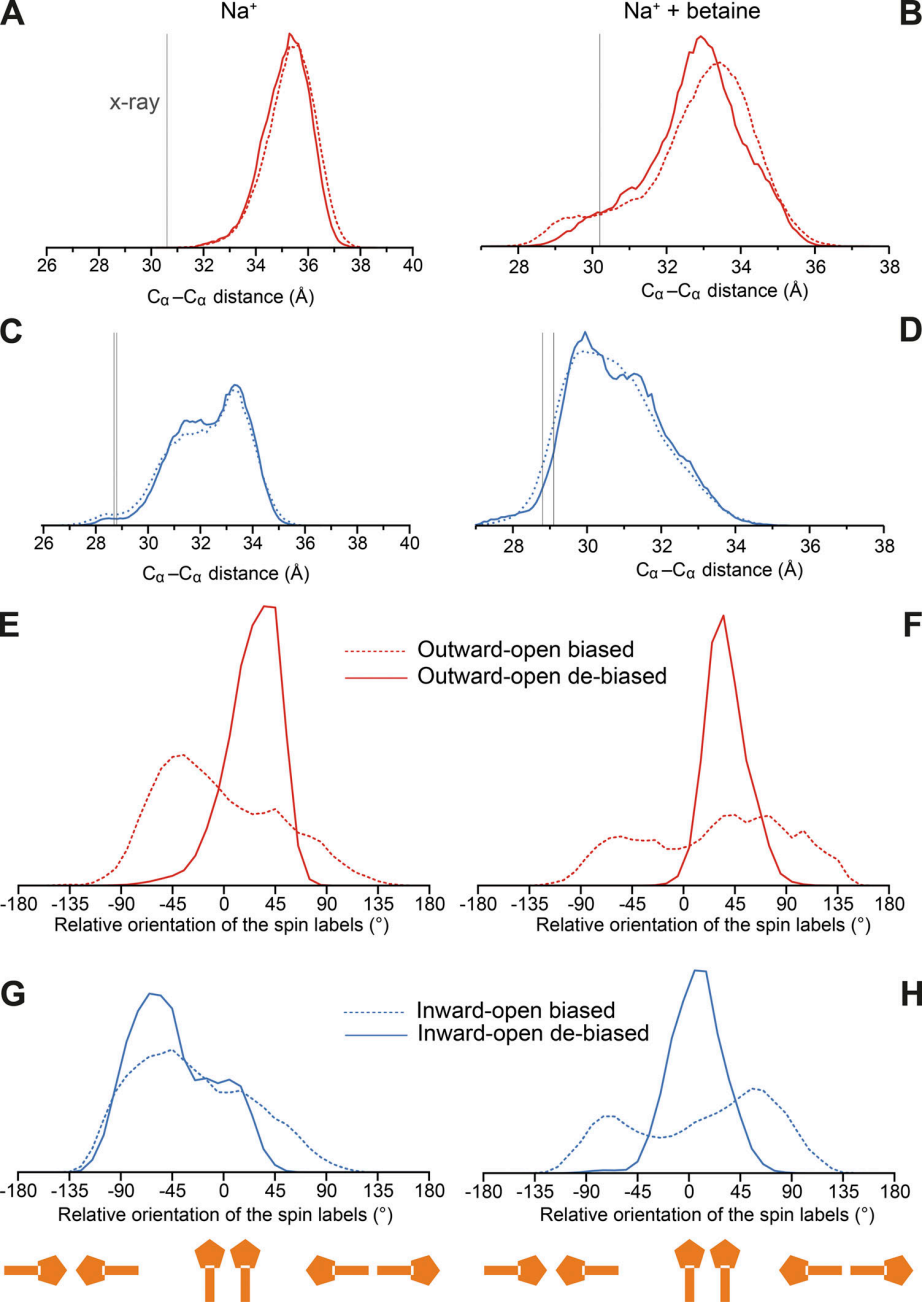
**Analysis of backbone distances and spin-label orientations**. Distributions of Cα–Cα atom distances (A–D) and spin-label side chain orientations calculated for the biased trajectories (dashed lines) and after de-biasing those same trajectories (solid lines; E–H). Simulations were performed with two sodium bound (left panels) or with two sodium ions plus one betaine molecule bound (right panels). Data were obtained for simulations of BetP in outward- (PDB accession no. 4LLH chain A, red), or inward-open conformations (PDB accession no. 4C7R, blue). **(A–D)** Reference x-ray structure data (vertical gray lines) are taken from Table S2. **(E–H)** The angle was computed between the axes connecting the backbone Cα and nitroxide N atoms in each spin label; negative numbers indicate that the probe rings point toward one another, whereas positive numbers indicate that the probes are oriented in opposite directions.

The RMSD analysis demonstrates that the biases applied during these simulations do not result in the protein adopting a different conformational state. What, then, is the additional work that is required? To address this, we attempted to break down the work into its contributing factors, based on the probability distributions of specific characteristics of the molecular system. We then compared the distribution that was obtained in the EBMetaD-based trajectory with the distribution obtained for a trajectory in which the bias had been removed (debiased; see Materials and methods), which provides a measure of the work performed to impose (or prevent) that feature, since larger differences in the distributions indicate more work was required (Hustedt et al., 2018). We first asked whether the distance between the backbone atoms was being affected. In fact, only a very small amount of work is applied to the distance between the backbone atoms (Fig. 7 A), even for the simulations in the presence of betaine. Note that the range in the Cα–Cα distances due to thermal fluctuations is between 4 and 7 Å, depending on the system, even after reweighting the distribution without the applied bias (Fig. 8, A–D).

If the backbone to which the probe is attached readily samples a range that is compatible with the experimental distribution in all conformational states, then what determines the differences in distance distribution? The obvious next candidate is the orientation of the spin labels (example conformations of which are shown in Fig. 1, C–F). We therefore asked how much work was done to alter their relative orientations. A substantial amount of work was required, up to ∼2.3 kcal/mol depending on the starting structure (Fig. 7 B). In particular, the probe orientation appears to be a major factor in the difference between the outward- and inward-facing states. For example, in the Na^+^-bound outward-open conformation, the bias encourages the probes to sample more conformations in which they point toward one another; after removing the bias, they tend to point away (Fig. 8, E–H). Note, however, that the magnitude of this contribution depends on the presence of the substrate betaine (Fig. 7 B).

Altering the relative orientations of the spins may require the adoption of rare, less inherently favorable side-chain dihedral angles. Indeed, analyzing the changes in all five backbone dihedral angles of the two spins suggests a nonnegligible contribution of the bias to altering the dihedral angles (Fig. 7 C). This contribution tends to be higher for the outward-facing than for the inward-facing conformations. All five dihedral angles contribute, but the largest component involves the χ_2_ angle (Fig. 7 D).

The relative spin orientation contribution alone does not account for the entirety of the work done to match the experimental distributions. We also tested how much work was associated with exposing the probe to solution. This value was <0.06 kcal/mol in all four systems, indicating a minor contribution. Finally, we also asked whether specific interactions between the probes and neighboring residues were associated with a large contribution to the work. From visualization of the trajectories, we identified a number of contacts that occur between the label and the rest of the protein. For any given set of interacting residues, the magnitude of the work depended on whether the protein was inward- or outward-facing (Fig. 7 E). For example, the probe attached to position 516 interacts with residues 85–93 in TM −1′, but only in the outward-open conformation with Na^+^ bound. Interestingly, for the simulation with betaine, no substantial work was associated with either forming or breaking those same interactions (Fig. 7 E). Overall, these results illustrate how complex the contributing factors can be for the conformational distribution of any given pair of spins in a given conformation of the protein.

## Discussion

The increasing availability of a range of structural and biophysical information for proteins such as BetP has revolutionized the membrane transport field, but has also presented challenges, in that different sources of data often appear to be incompatible or not clearly consistent. In this context, novel strategies to evaluate and integrate different kinds of experimental data with atomistic, dynamic representations of these proteins and their environments have the potential to drive further mechanistic insights. In the case of BetP, for example, the small distance changes implied by comparison of the outward- and inward-open crystal structures cannot be readily reconciled with the broad distance distributions obtained for spin labels attached to the protein, and the introduction of a major new peak when the substrate betaine is added to the solution. However, atomistic molecular simulations of the spin-labeled protein, based on the enhanced-sampling technique EBMetaD, provide the molecular detail and thermodynamic framework required to resolve these discrepancies. Here, we illustrate how the conformations of BetP present in proteoliposomes during EPR measurements may be ranked according to their compatibility by comparison of different simulation setups. We focused on the compatibility of two major conformational states, namely with the extracellular pathway closed or open. For simplicity, each state was simulated separately, testing the scenario that each experimental distribution could reflect a single major state. In the future, it may be of interest to test the compatibility of mixtures of states, interchanging states, or additional states, using multiple-replica approaches, as proposed previously (Hustedt et al., 2018). Nevertheless, even within the limited framework of this proof-of-principle study, it is of interest to consider the implications of the results from the perspective of sodium-coupled betaine uptake.

### Possible implications for BetP transport mechanisms

With the caveat that they were obtained for a single pair of probes on the periplasmic surface of BetP, our results suggest that the inward-facing (or outward-closed) conformation may be dominant in the presence of sodium and no betaine. Such a conclusion would be nontrivial, given that the physiological role of BetP is to capture betaine from the extracellular solution, which implies a preference for the outward-facing state in the presence of sodium, like LeuT (Kazmier et al., 2014b; Tavoulari et al., 2016). On the other hand, these observations for BetP would be consistent with EPR measurements on two other sodium-coupled transporters, Mhp1 (Koshy et al., 2013; Kazmier et al., 2014a) and vSGLT (Paz et al., 2018), and with the fact that BetP crystallizes preferentially in inward-facing conformations in saturating sodium concentrations (Perez et al., 2012). Clearly, a complete assignment of the state of BetP would benefit from analysis of additional pairs of probes on different regions of the protein. It should also be noted that the measurements were performed in proteoliposomes with equal concentrations of substrates on either side of the membrane. The reason behind this choice is that the orientation of BetP in liposomes is unknown, and therefore this protocol ensures that all proteins will experience the same environment. Consequently, we cannot rule out the possibility that BetP would instead prefer outward-open states if an inwardly oriented sodium gradient were applied.

A different picture was obtained for the data measured in the presence of betaine. Neither the inward- and outward-facing conformations of BetP individually satisfied the distribution obtained under these conditions. It is possible that the data reflect a third conformation, such as the fully occluded state, not simulated here. However, the targeted experimental distance distribution contains two peaks in the range of intraprotomer interactions, and therefore it seems likely that the experimental ensemble comprises a mixture of two or more states. As mentioned, future studies of the behavior of BetP in the presence of substrates could explore these possibilities using a multiple-walker strategy.

### Sensitivity of the methodology to the experimental distribution

In this study, the EBMetaD methodology was used to reproduce distributions that had been obtained from PELDOR time traces using Tikhonov regularization. However, that conversion does not result in a unique solution, and therefore it is possible that the magnitude of the biasing work applied during the simulations would differ if a different solution were to be considered. A similar concern relates to the post-processing of the distributions to remove interprotomer contributions. Recomputing the biasing work that is required to enforce the experimental distributions on the periplasmic pair only for distances <37 Å reproduced the trends observed for the entire distribution (Fig. 5 B). Thus, although the exact value of the work applied may vary to some degree, the overall strategy appears not to be sensitive to the precise details of the distribution. By extension, the approach is probably also not sensitive to variations in the solution of the Tikhonov regularization procedure. Nevertheless, we note that recent developments in EBMetaD provide for inclusion of the uncertainty due to the transformation (Hustedt et al., 2018), or even applying the echo decays from the EPR measurements directly (Marinelli and Fiorin, 2018). We expect that these methodologies will produce similar conclusions while reducing uncertainties due to data processing. In principle, the direct use of echo decays is most directly informative. However, in cases of oligomeric states in which long-range contributions may need to be excluded, the use of filtered distance distributions (with or without uncertainty bars) would seem still to be a reasonable strategy.

In conclusion, this study illustrates the application of EBMetaD simulations as a technique to facilitate an integrative, molecular-level interpretation of structural and spectroscopic data for membrane proteins. In particular, this technique can provide a clear view of whether the data can be well described by one of a number of available structures, or whether no one structure alone is compatible with the data.

## Supplementary Material

Supplemental Materials (PDF)

EBMetaD command files
