# Supplementary material for: Interpretation of spectroscopic data using molecular simulations for the secondary active transporter BetP
Source: J Gen Physiol. 2019 Mar 4;151(3):381–94. doi: 10.1085/jgp.201812111 (PMC6400524; doi:10.1085/jgp.201812111)
Supplement: Supplemental Materials (PDF) [file JGP_201812111_sm.pdf]

Supplemental material

Leone et al., https://doi.org/10.1085/jgp.201812111

Processing the PELDOR data for multi-spin contributions

In the configuration of two spin labels on each protomer, BetP contains six spin labels per trimer. Aside from the large number (i.e., 15) of possible spin–spin interactions, the presence of multiple labels also raises the possibility of more than two spins coupling with each other. That is, a three (or more) spin system could not only lead to the expected three distances, but also to additional ghost distances due to trigonometric function with a non-linear relationship. These ghost distances would be due to larger (artificial) frequency, and therefore the ghost peaks would show up at shorter distances than expected. The probability of observing such ghost distances depends on the modulation depth of the signal, which in turn depends on the power of the applied microwave. While more "modern" Q-Band spectrometers with high 150-W microwave power could result in depths of 50% or more, we obtained data with relatively low modulation depth values (~20%), which helped to reduce multi-spin signals. We also reduced the potential contribution of ghost peaks by power scaling of the recorded PELDOR time traces (1). Specifically, the normalized time trace is scaled by the power of $1/(n\text{-}1)$, where the contributing number of spins is n. In practice, this was accomplished by analyzing the PELDOR time traces using the "ghost spin" option in the DeerAnalysis software, rescaling them by the power of 1/5, assuming six spins.

Assessing the contribution of multi-spin dipolar couplings

To assess the likely contribution from multi-spin dipolar couplings, we used MMM 2013 to model the rotamer states of the MTSSL spin label on positions 450 and 516 of an x-ray structure of the BetP trimer (PDB accession no. 4DOJ; Fig. 1). First, to identify the contributions from intra- and interprotomer spin label pairs that would occur in the absence of multi-spin coupling, we modeled each pair of spin labels and computed the corresponding spin–spin distances, as well as the corresponding predicted PELDOR time traces for various combinations thereof. Second, we generated models of the BetP trimer containing all six spin labels, for which we computed the corresponding distance distributions and the PELDOR time traces, including all multiple spin effects that are theoretically possible. The latter predicted PELDOR trace should be directly comparable with the experimental data. To identify ghost peaks in the MMM 6-spin distance distribution, the predicted PELDOR time traces were then analyzed with DeerAnalysis to reverse engineer a distance distribution, using the same power-scaled approach used for the experimental data. Specifically, the simulation assumed a modulation depth of 22% and, prior to applying Tikhonov regularization, the time traces were rescaled to the power of 1/5, to reduce ghost effects.

The MMM-simulated PELDOR distance distributions and time traces are shown in Fig. S1. Importantly, the complete distance distribution of the MMM six-spin structural ensemble (Fig. S1 A, black) is very similar to the reverse-engineered distribution from a simulated PELDOR time trace in which all multiple spin effects were taken into account (Fig. S1 A, cyan). In particular, when incorporating the multi-spin effect, no additional peak in the distance distribution was found at short distances, i.e., shorter than the intraprotomer distances Fig. S1 A, red). We note that although some variation is visible at larger distances, such effects would be negligible for time traces containing noise at levels encountered in typical experiments.

EBMetaD command files are provided as a separate PDF file.

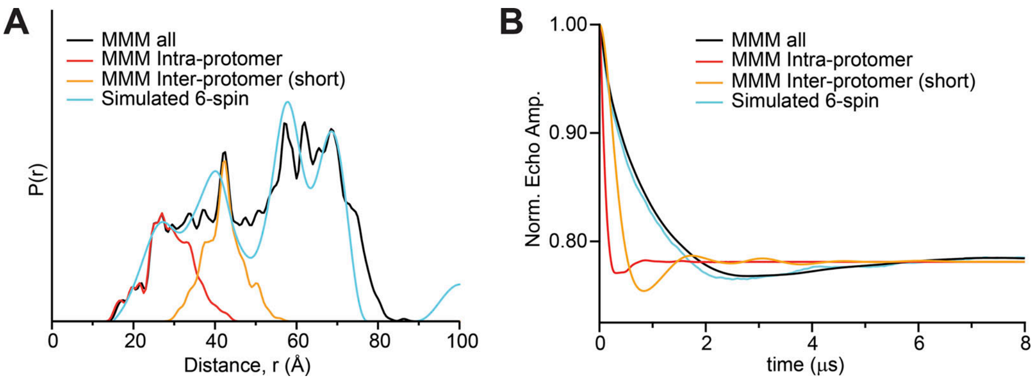

Figure S1. **Simulated PELDOR data examining the likelihood of ghost peaks due to multi-spin effects. (A)** Distance distribution extracted from predicted conformational ensembles of BetP spin labels at positions 450 and 516 in a trimer (PDB accession no. 4DOJ). The distances were estimated using MMM with the "R1A_298K_xray" rotamer library. We then extracted two-spin distances for all predicted two-spin distances (black), for all pairwise distances within a protomer (red), and for the shortest distribution of pairwise distances between two probes in different protomers (orange). The simulated six-spin distance distribution (cyan) was reverse engineered from the predicted PELDOR time trace shown in B using DeerAnalysis with "ghost spin" scaling of six spins. The Tikhonov regularization was performed with a smoothing parameter of 1,000. **(B)** PELDOR time traces predicted from the MMM spin label distributions shown in A, assuming either six spins (cyan), or only two-spin contributions (black, red, and orange).

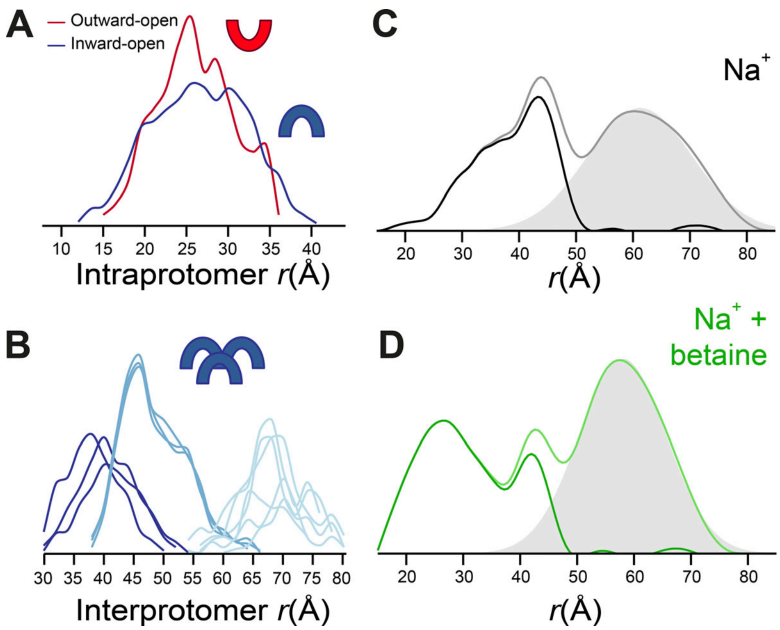

Figure S2. Spin-label distance distributions predicted by sampling spin-label rotamers on static crystal structures, and filtering of long-range peaks. Spin-label rotamer library–based predictions were conducted using the MMM package (Polyhach and Jeschke, 2010; Polyhach et al., 2011; Jeschke, 2012) for individual protomers in one of three conformational states **(A)**, or a trimer in the inward-facing state **(B)**. Probes were attached periplasmic positions G450 and S516. Lines represent the distances obtained for all rotamers of a single pair of labels. In B and D, different shades of blue highlight distinct populations observed at different distance ranges. **(C and D)** Filtering out of intraprotomeric distances from the periplasmic spin label distance distributions measured in the presence of either 500 mM NaCl (C), or 300 mM NaCl and 5 mM betaine (D). Based on the spin-label rotamer predictions (A and B), the peaks observed at ~60 Å must represent coupling between probes on adjacent protomers. Therefore, we subtracted this peak from the PELDOR-derived distance distribution for use with simulations of the BetP monomer. Specifically, a Gaussian function centered at this peak (shaded surface) was subtracted from the experimental data (darker lines) to obtain the final distribution (lighter lines) shown also in Fig. 2 D.

Table S1. **Primer sequences**

**Primer**	**Sequence (5′ to 3′)**
G450C$_{s}$	5′-CTTCATGCACTTCCAGGTTGCCAAATCATGGGC-3′
G450C$_{as}$	5′-GCCCATGATTTGGCAACCTGGAAGTGCATGAAG-3′
S516C$_{s}$	5′-GGTGACAATGCCTTGTGCAACTTGCAAAACGTCACC-3′
S516C$_{as}$	5′-GGTGACGTTTTGCAAGTTGCACAAGGCATTGTCACC-3′

Exchanged codons are underlined. Sense ($_{s}$) and antisense ($_{as}$) primers are listed.

Table S2. **Reference distances in x-ray structures of BetP**

	**Na**	**Na + betaine**
Outward	30.6 (4DOJ_B)	30.2 (4LLH_AB)
Inward	28.7 (4C7R_AB)	28.8 (4LLH_C)
	28.8 (4C7R_C)	29.1 (4AIN_C)
		29.1 (4DOJ_C)

Distance (Å) between the backbone Cα atom of Gly450 and Ser516 in known structures of BetP. PDB accession nos. and chain identifiers are given in parentheses. In some cases, the value is identical in more than one chain (e.g., 4C7R chains A and B).

References

Jeschke, G. 2012. DEER distance measurements on proteins. *Annu. Rev. Phys. Chem.* 63:419–446. https://doi.org/10.1146/annurev-physchem-032511-143716

Polyhach, Y., and G. Jeschke. 2010. Prediction of favourable sites for spin labelling of proteins. *Spectrosc-Int J.* 24:651–659. https://doi.org/10.1155/2010/706498

Polyhach, Y., E. Bordignon, and G. Jeschke. 2011. Rotamer libraries of spin labelled cysteines for protein studies. *Phys. Chem. Chem. Phys.* 13:2356–2366. https://doi.org/10.1039/C0CP01865A
