# Supplementary material for: Interpretation of spectroscopic data using molecular simulations for the secondary active transporter BetP
Source: J Gen Physiol. 2019 Mar 4;151(3):381–94. doi: 10.1085/jgp.201812111 (PMC6400524; doi:10.1085/jgp.201812111)
Supplement: EBMetaD command files [file JGP_201812111_TextS1.pdf]

Supporting Information: EBMetaD command files

COLVARS format

```
# output frequency
colvarsTrajFrequency 250

# selection of groups for defining a distance, with the center
# of mass of the N and O atoms from the spin label on 450 and
# the center of mass of the N and O atoms from the spin label on 516

colvar {
  # name of the distance colvar
  name dist_450_516
  distance {
    # selection of the N and O atoms of label on 450
    group1 {
      atomNumbers {
         6004 6005
      }
    }
    group2 {
      # selection of the N and O atoms of label on 516
      atomNumbers {
         6980 6981
      }
    }
  }
  ###boundary condition to prevent sampling of
  ###regions outside of the distribution
  # position of the lower and upper harmonic restraints
  lowerWall 20
  lowerWallConstant 100
  upperWall 50
  upperWallConstant 100
  # boundaries that defines the lowest and highest allowed value
  # used to define the grids of values for the colvar
  lowerBoundary    20
  upperBoundary    50
  # grid spacing
  width            0.5
}

metadynamics {
    name meta450_516
    colvars          dist_450_516
    # Gaussian parameters
    hillWeight       0.01
    hillWidth        0.5
    newHillFrequency 1000
    useGrids         on
    ebMeta           on
    # targeted input distance distribution file
    targetdistfile   deer_450_516.dat
    # number of equilibration steps
    ebMetaEquilSteps 100000
    # turn on the Gaussian reflection
    useHillsReflection   on
    # limits of the Gaussian reflection
    # outside these limits the bias force
    # is turned to zero
    reflectionLowLimit   20
    reflectionUpLimit    50
    writeHillsTrajectory on
}
```

PLUMED format

```
# output frequency
PRINT W_STRIDE 250

# Gaussian parameters
HILLS HEIGHT 0.01 W_STRIDE 1000

# selection of groups for defining a distance, with the center
# of mass of the N and O atoms from the spin label on 450 and
# the center of mass of the N and O atoms from the spin label on 516

DISTANCE LIST <g450> <g516> SIGMA 0.5 NOPBC

g450->
6004 6005
g450<-

g516->
6980 6981
g516<-

# targeted input distance distribution file
PROBRES CV 1 PROBFILE deer_450_516.dat

# boundary condition to prevent sampling of region outside of distribution
LWALL CV 1 LIMIT 20 KAPPA 100

UWALL CV 1 LIMIT 50 KAPPA 100

# limits of the Gaussian reflection applied at the boundaries
INVERT CV 1 REFLECTION 6 INVERSION 12 MAXHEIGHT 5 LIMIT1 20.0 LIMIT2 50.0

# zeros out any bias forces outside the interval
INTERVAL CV 1 LOWER_LIMIT 20.0 UPPER_LIMIT 50.0

ENDMETA
```
